# Artificial intelligence-based MRI radiomics and radiogenomics in glioma

**DOI:** 10.1186/s40644-024-00682-y

**Published:** 2024-03-14

**Authors:** Haiqing Fan, Yilin Luo, Fang Gu, Bin Tian, Yongqin Xiong, Guipeng Wu, Xin Nie, Jing Yu, Juan Tong, Xin Liao

**Affiliations:** Department of Medical Imaging, The Affiliated Hospital of Guizhou Medical University, 550000 Guizhou, Guiyang, China

**Keywords:** Glioma, Radiomics, Radiogenomics, MRI, Artificial intelligence, Machine learning

## Abstract

The specific genetic subtypes that gliomas exhibit result in variable clinical courses and the need to involve multidisciplinary teams of neurologists, epileptologists, neurooncologists and neurosurgeons. Currently, the diagnosis of gliomas pivots mainly around the preliminary radiological findings and the subsequent definitive surgical diagnosis (via surgical sampling). Radiomics and radiogenomics present a potential to precisely diagnose and predict survival and treatment responses, via morphological, textural, and functional features derived from MRI data, as well as genomic data. In spite of their advantages, it is still lacking standardized processes of feature extraction and analysis methodology among different research groups, which have made external validations infeasible. Radiomics and radiogenomics can be used to better understand the genomic basis of gliomas, such as tumor spatial heterogeneity, treatment response, molecular classifications and tumor microenvironment immune infiltration. These novel techniques have also been used to predict histological features, grade or even overall survival in gliomas. In this review, workflows of radiomics and radiogenomics are elucidated, with recent research on machine learning or artificial intelligence in glioma.

**Supplementary Information:**

The online version contains supplementary material available at 10.1186/s40644-024-00682-y.

## Highlights

AI-based radiomics and radiogenomics aim to provide aid in diagnosis and prediction with higher accuracy.

The introduction of MRI-based radiomics and radiogenomics analyses represents a non-invasive and cost-efficient adjunct tool that can extract quantitative information to augment clinical decision making.

Radiomics and radiogenomics present a potential to precisely diagnose and predict survival and treatment responses.

**Supplementary Information:**

The online version contains supplementary material available at 10.1186/s40644-024-00682-y.

## Background

### Epidemiology of glioma

Glioblastoma (GBM) and other central nervous system (CNS) tumors are among the most lethal tumors with substantial morbidity and mortality [1]. According to the latest CBTRUS Statistical Report, malignant brain and other CNS tumors resulted in 83,029 deaths due to between 2014 and 2018 in the United States [2], with a mortality rate of 16,606 deaths per year or 4.43 per 100,000. The 5-year relative survival for all malignant brain tumors combined increased between 1975 and 1977 and 2009–2015 from 23 to 36% [1]. GBM is the most common malignant primary brain tumor with a median survival of less than 2-year [3]. However, due to few advances in early diagnosis and efficacious therapy during the past four decades, less gains among older patients contributes to a higher burden of healthcare. Latest advances in genomic technology have contributed to a better understanding of pathology, molecular mechanisms and markers underlying GBM [4]. In the 2021 WHO update for CNS tumors, the classification of diffuse gliomas was redefined by integrating prognostically and biologically relevant genetic alterations with traditional histopathological assessment [5, 6]. Diffuse gliomas are broadly divided into lower grade glioma (grade II and III) and glioblastoma (grade IV) based on traditional histological grading. Both diffuse lower grade gliomas (LGGs) and glioblastomas (GBMs) are further subdivided into IDH-mutant and IDH-wildtype based on the presence or absence of isocitrate dehydrogenase gene 1 or 2 (IDH1/2) mutation. For IDH-mutant LGGs, the oligodendroglial or astrocytic lineages are defined based on the presence or absence of whole arm codeletion of chromosome arms 1p and 19q (i.e., 1p/19q codeletion). Under the new classification scheme, oligodendrogliomas (ODG) are defined by the presence of both IDH1/2 mutation and 1p/19q codeletion. IDH-mutant astrocytomas lack 1p/19q codeletion, and typically harbor mutations in ATRX and TP53 genes. In circumstances of incomplete or inconclusive genetic testing, the WHO assigns the “not otherwise specified” (NOS) label to oligodendrogliomas, astrocytomas, oligoastrocytomas, and glioblastomas. Other than in cases of incomplete/inconclusive molecular characterization, the diagnosis “oligoastrocytoma” is avoided. This integrated tissue-based molecular and histological characterization is the gold standard for classifying diffuse gliomas.

### Intratumoral heterogeneity in MRI diagnosis for GBM

In the past decade, critical advances in understanding the molecular biology underlying GBM have been obtained, but few has been translated into improved diagnosis and prognosis for patients [7]. GBM reveals noticeable intratumoral heterogeneity, which perplex clinical diagnosis and therapy [8]. Though conventional contrast-enhanced MRI techniques are incapable of perceiving this kind of heterogeneity, advanced MRI tools and PET imaging can present a wide range of image features to reflect physiologic and biophysical characteristics and improve the accuracy of MRI diagnoses for GBM. Integrating these advanced tools can be beneficial for screening histologically distinct lesions for surgery and radiotherapy, evaluating the regional heterogeneity of tumor and predicting therapeutic reaction after standard adjuvant therapy. The limitations in current imaging techniques offer an opportunity for radiomics and radiogenomics to augment the capabilities of MRI imaging.

### MRI radiomic features for survival prediction in GBM

Recent studies have shown that MRI radiomic features combined with genetic alterations and gene expressions could provide deeper insight to the classification of GBM subtypes and survival prediction [9–12]. For instance, presurgical MR images of 75 GBM patients with genomic data from the Cancer Genome Atlas (TCGA) portal were evaluated for tumor size, location, and morphology via a standardized imaging feature set, which demonstrated the capability of MRI imaging features in reproducibly characterizing patient survival [9]. Similarly, to assess the diagnostic and prognostic accuracy of MRI textural features obtained from contrast-enhanced T1-weighted (CE T1W) MRI sequences in GBM patients before the treatment, a training cohort of 165 patients from local institutions and an independent validating cohort of 51 patients from TCGA were included [11]. The surface-derived imaging biomarkers derived from CE T1W MRI is a sensitive predictor for survival of GBM patients, which might be helpful in classifying patients before resection surgery. A cohort of 404 GBM patients were recruited to investigate whether MRI imaging features obtained from pretreatment volumetric postcontrast T1W MRI and clinical information could predict survival of GBM patients [10]. Based on morphological features, a linear prognostic model predicted survival of GBM patients with high accuracy. In a retrospective review of 97 patients with multifocal glioblastomas, a prognostic model incorporating MRI imaging features on pretreatment volumetric postcontrast T1W MRI classified patients into different subgroups with variant survival [12].

### Artificial intelligence-based radiomics and radiogenomics for gliomas

Artificial intelligence (AI), an emerging branch of computer science, including “machine learning” and “deep learning,” are becoming important adjunct research tools in radiomics. With the help of sophisticated computational algorithms and training models, it is now possible to “data mine” hidden information from large clinical and imaging datasets. Machine learning belongs to a category of artificial intelligence, enabling a computer to learn from “training” data in existence beyond being explicitly programmed for predicting novel data, which focuses more on generating hypothesis rather than driving hypothesis [13]. Machine learning methods are mainly classified as unsupervised as well as supervised learning. Unsupervised learning provides no labels and applies features for discovering categories (natural groupings) of data. Oppositely, for supervised learning, datasets with labeled data (classes) are utilized as training for generating models that map features to the categories. Deep learning belongs to a subcategory of neural network-based machine learning that contains massive layers, especially applicable to images and videos. Deep learning aims at stimulating how neurons work as well as connect neurons to each other into a multilayer neural network, which contributes to the revolution of this field of imaging analyses, in which training on numerous digital images enables to test objects in images more favorable compared to human intelligence. Deep learning mainly comprises convolutional neural network (CNN), recurrent neural network (RNN), long short-term memory (LSTM), etc. Among them, CNN is the latest technology in the field of imaging analyses.

Recently, the research field of medical imaging has grown exponentially, with a great number of advanced analyzing tools and available datasets, which have boosted the substantial development of radiomics and radiogenomics [14]. It is foreseeable that conversion of digital images to high-throughput quantitative imaging features will eventually become routine clinical practice, and assist better clinical decision making, particularly in the care of GBM patients.

## Methodology of radiomics and radiogenomics

### Pipeline of radiomics

**Fig. 1:**
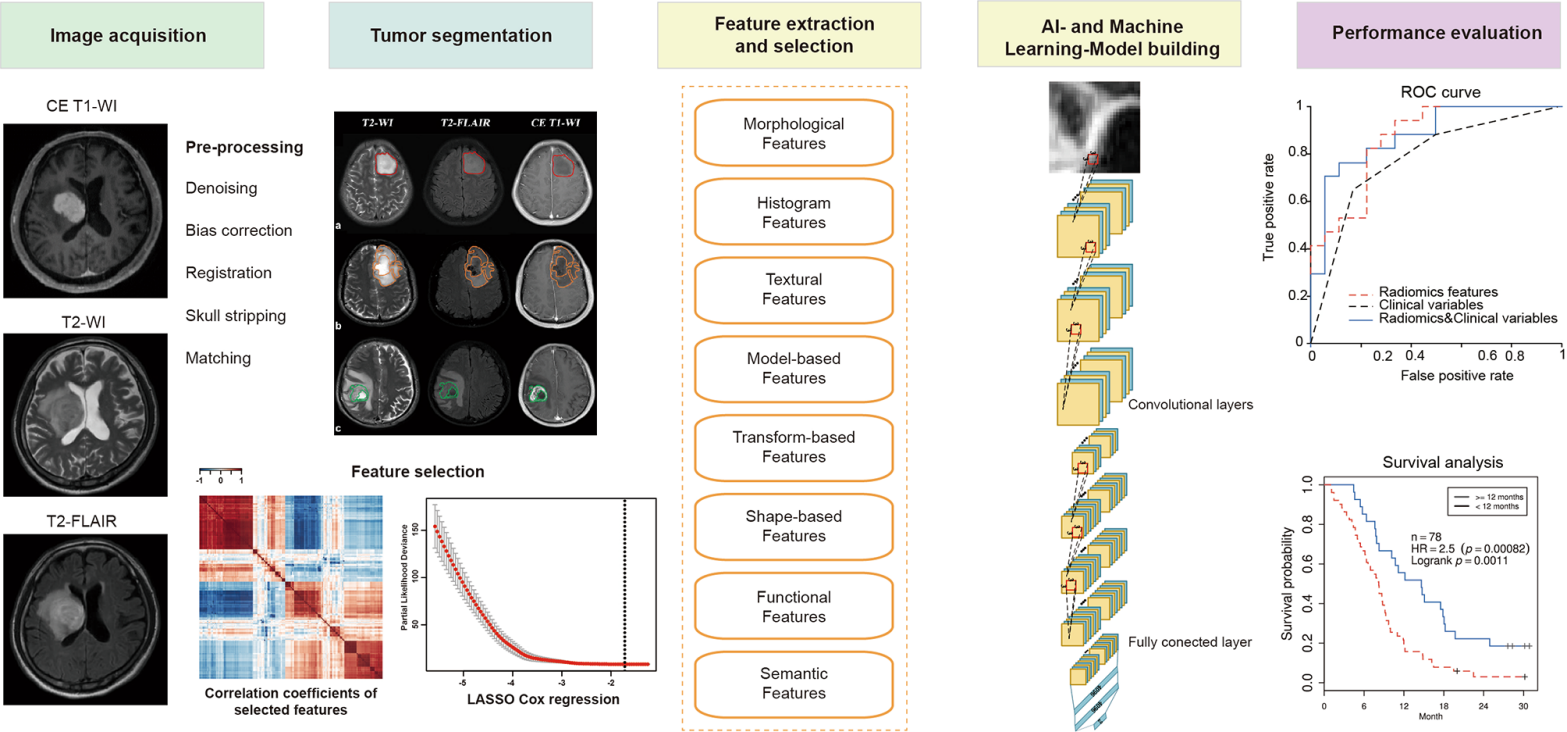
Pipeline of the general processing steps for radiomic studies. The flowchart presented the major processing steps needed for analysis of radiomic features from MRI in glioma. After skull stripping and artifact removal (bias field, noise, etc.), acquired MRI images are subjected to standardization and segmentation to extract regions of interests (ROIs). Radiomic features are then extracted from the image masks of ROIs via conventional radiomics or deep-learning approaches. After selecting relevant features, advanced statistical analysis is performed to classify and correlate radiomic features, involving machine/deep-learning methods for feature selection, classification, and cross-validation. Finally, endpoints are predicted to evaluate the models, such as patient’s survival, genomics, response to therapy, subsequent location of recurrence, or tumor micro-environment

Radiomics is an emerging application of advanced statistical methods in mining of high-throughput quantitative features from clinical medical imaging, in order to enhance predictive accuracy for diagnosis and prognosis models, especially within oncology [15–17]. These image features are being combined with clinical, genomic, transcriptomic and proteomic features to improve reproducibility [18]. Application of radiomics in oncology, including gliomas, usually refers to either classification tasks, such as benign vs. malignant, or prediction of clinical outcomes, such as overall survival. The major hypothesis of radiomics is that these predictive models can potentially improve diagnostic, prognostic, and predictive accuracy. As shown in Fig. 1, the radiomics methodology can be divided into five consecutive processes: (a) image acquisition, (b) region of interests (ROIs) segmentation, (c) feature extraction and screening, (d) model building and training, (e) model validation. As radiomics is an immature research field, each of the above processes poses its own challenges to be overcome.

The pipeline of radiomics is similar to any image recognition modality and corresponds to the workflow of machine learning or deep learning: images are input for an extractor of radiomic feature, and then a modeling process is used to map the radiomic features to the classification objectives (e.g., survival and subgroups) [19]. This pipeline makes each process highly dependent on the methods chosen by the previous steps. Hence, there are several limitations in each process. For ROIs or lesions, a great number of radiomic feature candidates can be extracted and screened out, which either variability most efficiently reveal data or fit a specific prediction model best [20]. Rather than screening out predefined set of features, deep learning pipeline combines feature selection and model construction directly to further improve goodness of fit and prediction accuracy.

Manual segmentation of medical imaging is a time-consuming work in routine radiotherapy to identify anatomical structures of lesions, which determines the efficacy and safety of therapeutic regimens [21]. However, inter- and intra-observer variability can significantly influence the accuracy of manual segmentation, which may further impair the quality of subsequent radiomic analyses and radiotherapy treatment choices. Automatic segmentation based on machine learning algorithms attempts to address this challenge [22], benefiting from advances in visual recognition and machine learning.

After feature extraction, feature selection is an important issue for subsequent analyses. Several algorithms have been proposed to select a subset of optimal features that are closely related to the presumptive outcomes, including minimum redundancy maximum relevance (mRMR) algorithm [23], sequential forward floating selection [24], and the multiview principal components analysis (Multiview-PCA) [25]. Feature selection aims to restrain potential overfitting connected with the high dimensionality of the radiomic features. Once optimal features are screened out, machine learning classifiers and AI algorithms are applied to constructed predictive models [26, 27]. However, a common defect for the application of these tools is the lack of normative paradigm and the high variability.

### Radiomic feature classes

Radiomic features can be generally classified into morphological, histogram, textural, model-based, transform-based, shape-based features [20]. For detailed explanations of radiomic features and corresponding equations, a reference manual from the Image Biomarker Standardization Initiative is recommended [28].

#### Morphological features

Morphological features, which aim to reflect the topology of tumor lesions, can be classified into global and local features [29, 30]. Global features quantify the contour of tumor lesion via measurements including roundness, perimeter, diameters of major and minor axes, as well as elongation factor. Local features refer to the surface curvature attributes of iso-surfaces, involving degrees of curvedness and sharpness.

#### Histogram features

Histogram features usually refer to the global gray-level histogram, including gray-level mean, maximum, minimum, variance, and percentiles. Other histogram features involve skewness, kurtosis, entropy and uniformity.

#### Textural features

Based on statistical and structural techniques, textural analyses evaluate the spatial distribution of grey values via local features at each image point and the relevant statistics, as well as identify structural primitives and the corresponding placement rules [31, 32].

Absolute gradient, as the simplest textural features, refers to the degree of gray-level intensity fluctuations, which involves gradient mean, variance, skewness, and kurtosis.

Gray-level co-occurrence matrix (GLCM), as a second-order gray-level histogram, involves entropy, uniformity, and contrast. GLCM reflects spatial relationships of pairs of voxels with predefined gray-level intensities in different directions, and with a predefined distance between the voxels.

Gray-level run-length matrix (GLRLM), which reflects the spatial distribution of runs of consecutive pixels with the same gray level in different directions and dimensions, involves fraction, long- and short-run emphasis (inverse) moments, and gray-level and run-length nonuniformity.

Gray-level size zone matrix (GLSZM) is computed for two dimensions (8 neighboring pixels) or three dimensions (26 neighboring voxels), involving fraction, large- and small-zone emphasis.

Gray-level distance zone matrix (GLDZM) is a ‘‘hybrid’’ of textural and morphologic features and involves small-distance high-gray-level emphasis.

Neighborhood gray-tone difference matrix (NGTDM) refers to the sum of differences between the mean gray level of a pixel or voxel and that of its neighboring pixels or voxels within an established distance. Key features of NGTDM involve coarseness, busyness, and complexity.

Neighborhood gray-level dependence matrix (NGLDM), which is based on the gray-level relationship between a predefined pixel or voxel and its neighborhoods, involves large- and small-dependence emphases, gray-level nonuniformity, and dependence uniformity.

#### Model-based features

Model-based features seek to reflect spatial gray-level information to portray objects or shapes.

#### Transform-based features

Transform-based features, involving Fourier, Gabor, and Haar wavelet transforms, aim to analyze gray-level patterns in a different space. Remarkably, wavelet transformation can be either used to generate radiomic features or to be a preprocessing step before texture analyses.

#### Shape-based features

Shape-based features interpret geometric properties of ROIs, including 2-demension and 3-demension diameters, axes, and their ratios, as well as compactness, sphericity and density.

#### Functional features

A key issue to the clinical application of radiomics is hard to explain the of biological interpretability of radiomic features [33, 34]. Hence, it is necessary to explore radiomic features with relevant biological interpretability. Functional radiomic features are a new cluster of biomarkers which indirectly reflect the underlying anatomy or physiology. Radiomics phenotypes of MRI, which were predicable for overall survival for GBM, were related to underlying physiological pathways such as cellular functions, tumor immune, tumor progression, and chemotherapy response [35]. T2-weighted MRI-based radiomic features in the prediction model for overall survival of gliomas were also related to immune response, especially tumor macrophage infiltration [36]. A combination of three radiomic features (volume-class, hemorrhage, and T1/FLAIR-envelope ratio), which was capable of predicting survival in GBM, were associated with molecular processes of growth and invasion pathways [37]. In a word, functional radiomic features provide a comprehensive insight into macroscopic picture of the tumor phenotype as well as its microenvironment.

#### Semantic features

Semantic features are descriptive features from structural MRI to reflect the location, shape, and geometric properties of tumor lesion [33]. The Visually Accessible Rembrandt Images (VASARI) feature-set criteria were used to obtain qualitative volumetric parameters, including contrast enhancement, necrosis, and edema/invasion [38]. These volumetric parameters were useful for predicting survival in GBM patients. A similar study also demonstrated that semantic features from presurgical MR images combined with genetic alterations and gene expression subtypes could provide deeper insight to the underlying biologic properties of GBM subsets from the Cancer Genome Atlas (TCGA) [9]. Semantic features can also be combined with machine learning-based radiomic features to improve prediction of progression and survival [39–42].

### Pipeline of radiogenomics

Radiogenomics refer to the combination of radiomics-derived imaging signatures and molecular markers [43], which will be beneficial to stratifying patients based on their specific imaging and molecular characteristics in order to design patient-specific precise therapies, such as noninvasive and global assessment of GBM [44]. Radiogenomics offers a reproducible tool to provide more information of intratumoral heterogeneity, which will improve the prediction precision for tumor progression, survival and response to targeted therapies [45], especially beneficial for recurrent GBM. In addition, radiogenomics can indicate either how a specific genomic mutation might affect the imaging characteristics of the tumors, or whether there is a potential association between molecular biomarkers and radiographic phenotypes [45–47]. However, efforts are needed to improve the utility of radiogenomics in clinical decision making for patients with gliomas [16].

**Fig. 2:**
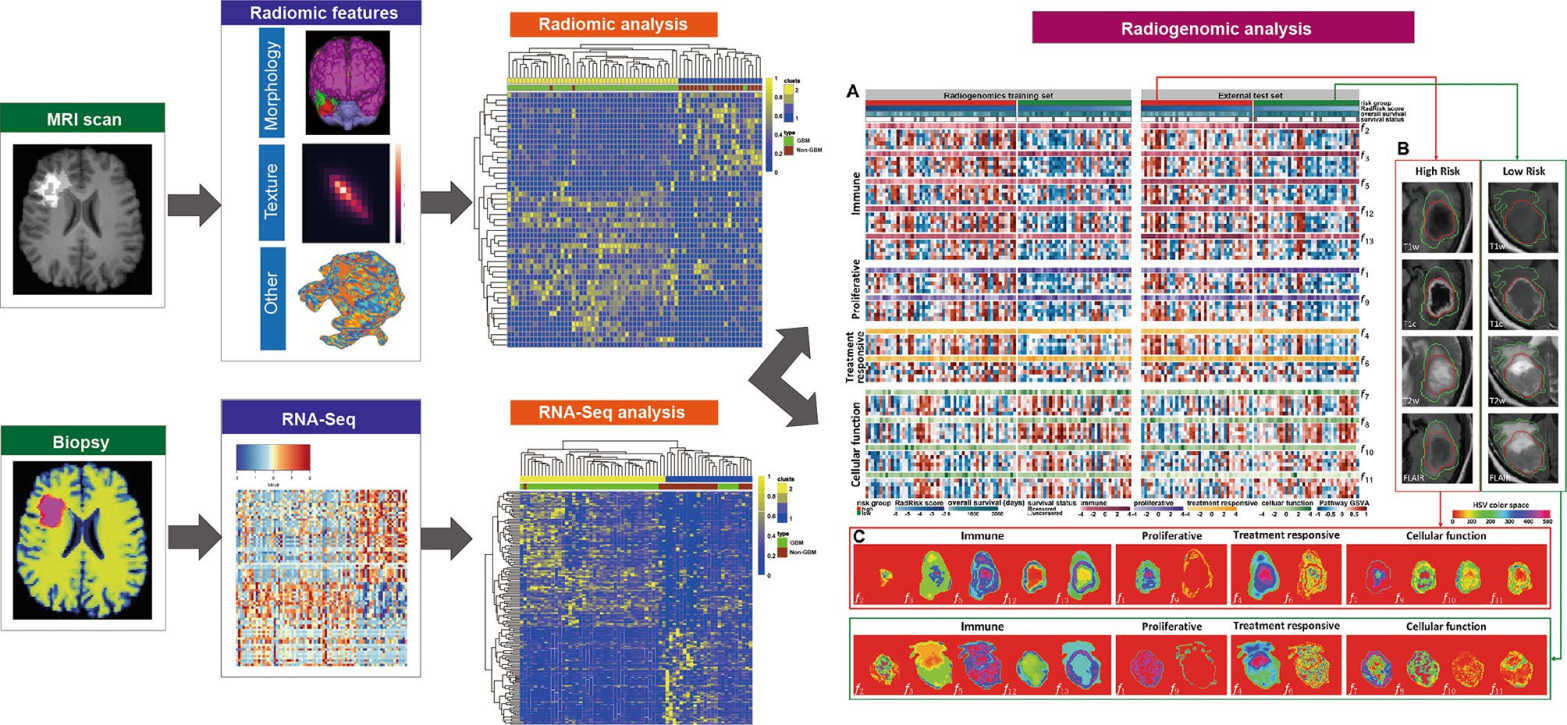
Pipeline of the general processing steps for radiogenomic studies. A typical radiogenomic analysis is usually conducted in four steps: (1) radiomic feature extraction and selection; (2) biopsy and RNA sequencing; (3) radiogenomics analysis and pathway identification; and (4) external validation. First, optimal MRI radiomic features are screened out to predict overall survival. Then, survival-relevant radiomic features are linked with co-expressed gene modules obtained by RNA sequencing. Furthermore, relevant pathways and key genes are identified to be able to annotate prognostic radiomic features. Finally, the reproducibility of prognostic radiomic-annotated pathways and key genes are validated in an external dataset

A typical radiogenomic analysis is usually conducted in four steps: (1) radiomic feature extraction and selection; (2) biopsy and RNA sequencing; (3) radiogenomics analysis and pathway identification; and (4) external validation (Fig. 2). In detail, optimal MRI radiomic features are screened out to predict overall survival. Then, survival-relevant radiomic features are linked with co-expressed gene modules obtained by RNA sequencing. Furthermore, relevant pathways and key genes are identified to be able to annotate prognostic radiomic features. Finally, the reproducibility of prognostic radiomic-annotated pathways and key genes are validated in an external dataset. The integration of multi-omic data accelerated the advent of radiogenomics in precise medicine for GBM. The critical object of radiogenomics is to associate genomic varieties and functional pathways with distinct radiomic phenotypes.

## Clinical applications

### Radiomics in glioma grading

Several studies have tried to assess the accuracy of MRI texture analysis in differentiating glioma subtypes and predicting survival. In a cohort of 95 patients with gliomas, with 27 low grade gliomas (LGGs) and 68 high grade gliomas (HGGs), MRI texture analysis was assessed to grade cerebral gliomas based on a filtration-histogram technique [48]. LGGs and HGGs were best classified via standard deviation (SD) at fine texture scale, with AUC of 0.91. Another similar cohort of 14 LGGs and 80 HGGs also demonstrated that LGGs and HGGs were best distinguished via mean of 2 mm fine texture scale with AUC of 0.90 [49]. The support vector machine-based recursive feature elimination (SVM-RFE) was used to screen out the optimal radiomic features derived from multiparametric MRI for LGGs and HGGs [32]. These features were then used to establish SVM classifiers to assess the grading efficiency. The accuracy was 96.8% for distinguishing LGGs from HGGs, and 98.1% for discriminating grades III from IV. Textural features from dynamic contrast-enhanced MRI (DCE-MRI) also presented a good performance in glioma grading [50].

### Radiomics in classifying molecular phenotypes of gliomas

As the most aggressive primary central nervous system malignancy, the clinical outcome of GBM has been revealed to vary depending on the extent of initial resection and response to chemo-radiation therapy with a median survival of 15 months [51, 52]. Radiomics and radiogenomics have been developed to investigate the associations between genetic phenotypes and MRI radiomic features in GBM [45], in consideration of numerous benefits, such as noninvasive and comprehensive evaluation of tumor lesion and treatment response. Numerous associations have been identified, which relate MRI radiomic features to underlying physiological characteristics [53], such as associations between hypo-intensity on pre-contrast T1-weighted MRI and necrotic tissue, as well as hyper-intensity on T2-weighted MRI and edema. In addition, quantitative radiomic features have shown significant associations with molecular and genomic phenotypes relating to cell proliferation and apoptosis. AI-based radiomics have also been applied to predict IDH genotypes in gliomas. A recent study tried to predict the IDH status of gliomas from MR imaging by applying a residual convolutional neural network to preoperative radiographic data [PMID: 29,167,275]. Similarly, an explainable recurrent neural network model based on DSC perfusion MRI was developed to predict IDH genotypes in gliomas [PMID: 31,127,834].

Recent studies have tried to explore the correlation between radiomic features and molecular phenotypes of gliomas. Based on a random forest model, radiomic MRI phenotyping was demonstrated to improve survival prediction when combined with clinical and genetic profiles [54]. To construct a model for classifying isocitrate dehydrogenase (IDH) status in gliomas based on multiparametric MRI, a total of 105 patients of grade II-IV gliomas were obtained with 50 IDH mutant and 55 IDH wildtype [55]. IDH genotypes could be discriminated by radiomics features with accuracy and AUC of 0.823 and 0.770, respectively. Based on the GBM subset from The Cancer Image Archive (TCIA) and a validation dataset from Japan, radiogenomic analysis reported a similar tendency in a machine learning classifier for IDH1/2 mutation prediction [56]. To detect the IDH1 mutation in LGG patients, 23 VASARI features from conventional multimodal MRI and 56 radiomic features from apparent diffusion coefficient (ADC) maps were extracted to establish a machine learning model via a random forest classifier, which obtained an AUC to 0.879 [57]. A radiomic model based on automated texture analysis and VASARI features was also used to predict the genetic status of IDH1 mutation and 1p/19q codeletion in LGGs [58]. Texture analysis was found to be more accurate than VASARI features.

Radiomic features were also used to determine the genetic profiles of ATRX, IDH1/2, MGMT and 1p19q in gliomas [59]. The BraTS 2019 pretrained DeepMedic network obtained a satisfying performance with AUCs of 0.979 for the prediction of the ATRX expression loss, followed by 0.929, 0.999, and 0.854 for the prediction of IDH1/2, 1p19q and MGMT, respectively. Furthermore, addition of MR diffusion to conventional MRI features significantly improved the diagnostic value in preoperative prediction of IDH1, MGMT, and ATRX in patients with glioma [60]. ^18^F-FET PET-MRI including MR fingerprinting also have been demonstrated to have the predictive potential of predicting the mutational status of ATRX, IDH1, and 1p19q, with AUCs of 85.1% for ATRX, 75.7% for MGMT, 88.7% for IDH1, and 97.8% for 1p19q [61]. A subgroup of 9 radiomic features from was screened out of 431 radiological features, which could predict the Ki-67 expression level in LGG patients with AUC of higher than 90% [62].

Compared with conventional radiomics, a sparse representation-based radiomics obtained a superior performance in differentiation of PCNSL and GBM and prediction of IDH1 mutant status [63]. In a cohort of 200 IDH1 wild-type GBM patients, an integrative model based on a panel of radiomic features, clinical profiles, and protein expressions (CIC, PIK3R1, FUBP1, p53, vimentin) obtained an AUC of 78.24% in predicting survival outcomes [64]. Based on pretreatment T2-weighted MRIs from 45 GBM patients, a deep neural network (V-Net) presented robust segmentation capability compared with manual segmentation; however, radiomic features from both manual and V-Net segmentation methods presented similar performances in IDH1 prediction [65].

Based on Haralick texture parameters derived from preoperative FLAIR sequence, the homogeneity parameter was demonstrated to be able to differentiate IDH mutated LGGs from IDH wild type LGGs [66]. However, the other Haralick texture parameters, such as energy, entropy, and inertia, could not discriminate LGGs with or without IDH mutation. Furthermore, tissue heterogeneity (homogeneity and pixel correlation) and FLAIR border distinctiveness (edge contrast, or EC) could optimally classify grade II/III gliomas by IDH status, which may give a hand for subsequent therapeutic choices [67].

Radiomic features, such as morphological, intensity, and textural features, were extracted from MRI of 32 IDH1-wild GBM patients and 7 IDH1-mutant patients to predict mutations in the IDH1 gene in GBM via a logistic regression classifier [68]. The accuracies of morphological and intensity features were 51% and 59% respectively, while that of textural features was significantly higher with an accuracy of 85%. To evaluate the potential of radiomics as a noninvasive predictor of 1p/19q codeletion status, 152 features were extracted from fluid-attenuated inversion recovery (FLAIR), T1-weighted images (T1WI) and post-contrast MRI of 47 LGG patients with IDH mutation [69]. Best classification was achieved via the Ensemble Bagged Trees classifier with AUC of 0.87. To recognize the best machine learning classifier for prediction of IDH mutation status in diffuse gliomas, 8 classical machine learning algorithms were assessed based on 704 radiomic features extracted from pre-operative MRI of 126 patients [70]. Random forest was found to be a promising classifier in predicting IDH mutation status with high predictive performance (AUC of 0.931). To establish a predicting model for IDH mutation status and 1p19q codeletion in glioma patients, radiomic features were extracted from preoperative MRIs of T1 contrast enhanced and T2-FLAIR sequences [71]. A random forest model for IDH mutation status obtained an AUC of 0.921 in the training cohort and 0.919 in the validation cohort from TCIA. The overall accuracy for three-way classification (IDH-wild type, IDH-mutant and 1p19q co-deletion, IDH-mutant and 1p19q non-codeletion) was 78.2%.

After semi-automatic segmentation of tumor lesions, radiomic features from pre-operative multiparametric MRI from 88 GBM patients were extracted [72]. IDH1 mutation status was determined by targeted sequencing and immunohistochemistry. Machine learning algorithms was demonstrated to be able to classify IDH1 mutation status in GBM patients with reliable accuracy.

Based on dynamic susceptibility contrast (DSC)-MRI from treatment-naïve gliomas, a random-forest model was applied to discriminate IDH mutation status via the extracted features, including shape, distribution and texture features [73]. Glioma patients were correctly classified by IDH mutation status in 71% of the cases.

### Radiomics in discriminating radiation necrosis, pseudoprogression and tumor recurrence

Contrast-enhancement at MRI after irradiation of GBM usually detects a new lesion, which leads to the challenge of differentiation between tumor recurrence and radiation necrosis induced by irradiation [74–77]. Pseudoprogression is a major clinical challenge after irradiation in GBM, which not only interferes with daily care but also disturbs the choice of therapeutic treatments [78]. However, traditional structural MRI has usually failed in discriminating tumor recurrence from radiation necrosis and pseudoprogression. Advanced imaging techniques, such as perfusion MRI, MR spectroscopy (MRS), or their combination, are expected to improve diagnostic accuracy. Despite availability of advanced imaging, radiomics were also regarded to provide complementary diagnostic information for traditional structural MRI and to help to differentiate tumor recurrence from radiation necrosis [79]. In addition, radiomics based on multiparametric MRI features and machine learning algorithms may be a promising non-invasive approach to differentiate radiation necrosis and tumor recurrence in resected GBM patients after chemoradiation [29, 80–82].

After surgical excision, radiation therapy with concurrent and adjuvant temozolomide-based chemotherapy were the current standard therapeutic schemes for GBM. Apart from tumor recurrence, radiation necrosis and pseudoprogression have also been recognized after chemoradiotherapy [83]. Specific MRI T1 post-contrast enhancement patterns were supposed to benefit differentiation between tumor recurrence and radiation necrosis in patients with high-grade gliomas [84]. Quantitative 3D shape features of T1WI and T2WI/FLAIR sequences in the enhancing lesions were also demonstrated to be able to more precisely reflect pathophysiologic variances across pseudoprogression and tumor recurrence [29].

Texture analysis of traditional MRI based on a SVM classifier to discriminate brain metastasis from radiation necrosis obtained a high diagnostic accuracy with AUC of 0.9 [85]. Co-occurrence of local anisotropic gradient orientations (CoLlAGe), a new radiomic feature derived from gadolinium-contrast T1WI, could reflect visually indistinguishable differences between benign and pathologic phenotypes on routine MRI, and might distinguish radiation necrosis and tumor recurrence of posttreatment lesions [86, 87]. A one-class-SVM classifier based on an 8-dimensional feature vector derived from multiparametric MRI features after alignment to post contrast T1WI could differentiate radiation necrosis from recurrent tumor with AUC of 94.39% [80]. To discriminate between radiation necrosis and recurrent tumor, textural analyses based on contrast-enhanced MRI (CE-MRI) obtained a diagnostic accuracy of 81%, while 83% for textural features derived from ^18^F-FET-PET [88]. After combining textural features of both CE-MRI and ^18^F-FET-PET, the diagnostic accuracy was improved to 89%.

### Radiomics in evaluating the efficacy of antiangiogenic therapy

Glioma stem cells promote angiogenesis via activating the transcriptional program mediated by hypoxia-inducible factors (HIFs), which limited the efficacy of antiangiogenic cancer therapies within hypoxic tumor microenvironment [89, 90]. Hypoxia, a typical characteristic of GBM, is regarded to result in chemoradiation resistance and related to unfavorable prognosis. To evaluate the efficacy of antiangiogenic therapy in differential clusters of GBM patients, radiomic features from preoperative and pretherapy perfusion MRI were extracted and unsupervised clustering was conducted to classify robust clusters of GBM patients [91]. Angiogenesis and hypoxia pathways were enriched in a subgroup of GBM patients with elevated perfusion features and significantly associated with a poor survival. Recently, a subset of MRI radiomic features, which were most informative of hypoxia enrichment score (HES) derived from the expression profile of 21 hypoxia-associated genes, was demonstrated to be able to discriminate GBM patients as short-term (STS) and long-term survivors (LTS) [92].

Bevacizumab, a monoclonal antibody to the VEGF, is the most widely used antiangiogenic agent for recurrent GBM. However, its treatment response varies from patient to patient, and effective biomarkers for patient selection are still not available. Radiomic features may provide prognostic value for survival and progression in patients with recurrent GBM after bevacizumab treatment [93]. Compared with clinical or volumetric covariates, multivariable analysis of radiomic features revealed a stronger ability in stratifications of OS and PFS. Before bevacizumab treatment, high-throughput MRI radiomic features were automatically extracted for a supervised principal component analysis to establish a prognostic model for predicting the therapeutic outcome to bevacizumab via PFS and OS [94]. This model successfully stratified patients into a low- or high- risk group of PFS and OS in both the discovery and validation sets. Based on 3,800 glioma and GBM patients across four relevant datasets, including CGGA and TCGA for RNA-Seq data, the Ivy Glioblastoma Atlas Project (Ivy-GAP) and TCIA for clinicopathological data, and The Clinical Proteomic Tumor Analysis Consortium Glioblastoma Multiforme (CPTAC-GBM) for proteomic data, radiogenomic analysis revealed that SOCS3 expression level was significantly correlated with radiographical features of perfusion imaging, and it could be a potential biomarker for predicting treatment response of bevacizumab in GBM patients [95]. In a cohort of 194 recurrent GBM patients treated with bevacizumab, radiomic features from pre-treatment T2 FLAIR and gadolinium-injected MRI along with clinicopathological data were used to predict OS and PFS via machine-learning algorithms [96]. Binary classification models successfully stratified the OS at 9, 12, and 15 months with the AUCs of 0.79, 0.82, and 0.87 on the training set and 0.78, 0.85, and 0.76 on the validation set, respectively. Similar results were also reported in another dataset from the 2019 BraTS challenge (210 patients) [97].

To determine whether texture analysis could improve the evaluation of response to bevacizumab in gliomas, MRI from 33 patients with HGGs before and after the treatment of bevacizumab were evaluated [98]. After bevacizumab treatment, lower edge contrast of the FLAIR hyperintense region was associated with poorer OS and PFS. Edge contrast cutoff significantly stratified patients for both OS and PFS. Hence, texture analysis based on edge contrast of the FLAIR hyperintense region could be a predictive indicator in patients with HGGs after treatment with bevacizumab.

### Radiomics and radiogenomics in survival stratification

Based on radiomic features from T2-weighted MRI of LGGs patients, a radiomic-based risk score was constructed and was used to stratify patients into low- or high-risk groups for overall survival [99]. Then, radiogenomic analysis further indicated that the risk score was related to biological pathways of hypoxia, angiogenesis, apoptosis, and cell proliferation. Similarly, radiomic features, derived from preoperative T2-weighted MRI in LGG patients from the Chinese Glioma Genome Atlas (CCGA) and TCGA, were found to be a good calibration for prediction of PFS [100]. These radiomic features were also significantly associated with the biological pathways of immune response, programmed cell death, cell proliferation, and angiogenesis.

Radiomic features from both T2WI and CET1WI in different genomic profile groups of GBM patients were analyzed to identify the potential imaging-molecular associations [101]. Major genomic profiles of GBM revealed a significant association with radiomic features. Radiomic features was revealed to be able to predict major genomic profiles and the prognosis of GBM patients. A combination of MRI imaging, miRNA and mRNA expression of 92 GBM patients from TCGA-GBM collection was able to significantly stratify survival in a statistically manner [37]. Radiogenomic analysis revealed that immune-associated pathways, such as natural killer cell activity and T-cell lymphocyte differentiation, as well as metabolism-associated pathways, including mitochondrial activity and oxidative phosphorylation, were underlying the survival characteristics. Sixteen three-dimensional (3D) textural heterogeneity features of post-contrast pre-operative T1-weighted MRI in GBM patients were extracted, including 11 run-length matrix (RLM) features and 5 co-occurrence matrix (CM) features [102]. Four RLM features and four CM features were revealed to be reliable predictors of survival. A novel set of texture features were generated through joint intensity characteristics of CE-T1 and FLAIR images in necrosis, active tumor, edema and invasion lesions of GBM patients [103]. Then, a random forest model was used to classify GBM patients into short or long survival subgroups. When combining these features with gene expression phenotypes, radiogenomic features obtained an AUC of 77.56%.

Based on the T1-weighted contrast-enhanced (T1W CE), T1W, T2-weighted (T2W), and fluid-attenuated inversion recovery (FLAIR) sequences from 119 GBM patients in TCGA, radiomic features reflecting tumor heterogeneity were extracted for each sequence, including the co-occurrence matrix, run-length matrix, and histogram features [104]. When a single sequence was used for predicting, the T1W-CE sequence obtained the highest AUC of 83.33%; when combining the four sequences, the predicting accuracy was barely close to that of T1W-CE sequence alone. Based on post-contrast T1W and T2 FLAIR MRI from 82 GBM patients, 5 sets of texture features were extracted to predict GBM molecular subtypes and 12-month survival status via a random forest model [105]. Radiomic analyses indicated that these texture features were reliable predictors for GBM molecular subtypes and survival status. A total of 45,792 radiomics features derived from multi-modality MR images were automatically extracted to predict OS of GBM patients [106]. Voxel size, quantization method and gray level were found to influence the predicting performance for prognosis of GBM patients. Texture features, tumor shape and volumetric features were extracted from MR images of 163 GBM patients [107]. After the feature selection via SVM-RFE, the predicting model obtained a promising accuracy in both the 2-class (short and long) and 3-class (short, medium and long) OS groups of 98.7% and 88.95%, respectively.

A recent radiogenomic analysis revealed associations of the MRI-based phenotypes with the signaling pathways in GBM [108]. In the poor OS group of male GBM patients, higher expressions of Laws energy features from the contrast-enhanced tumor lesions were detected, and aggressive pathways of cell adhesion and angiogenesis were found to be more enriched. However, in the poor OS group of female population, higher expressions of Laws energy features from the necrotic core were found to be significantly associated with pathways related to immune.

A deep learning framework using three different 3D convolutional neural network (CNN) architectures (W-Net, T-Net, and E-Net) was applied for brain tumor segmentation based on multimodal MRI from glioma patients [109]. A total of 4,524 radiomic features were extracted from segmented tumor regions for each subject, and then a decision tree and cross validation were used to screen out optimal features. A random forest model is trained to predict the overall survival of patients with a reliable accuracy in the classification of short-, mid- and long-survivors. A total of 348 manually extracted radiomic features and 8,192 deep features generated by a convolutional neural network (CNN) was used to predict overall survival in HGG patients [110]. After feature selection, an Elastic Net-Cox model was able to classify patients into long- and short-term survivors with satisfying performance.

## Discussion

### Current status and challenges facing clinical implementation

Conventional contrast-enhanced MRI usually fails to identify intratumoral heterogeneity, which is pronounced in GBM patients and interferences clinical diagnosis and therapy [8]. Through integrating advanced MRI techniques, radiogenomics based on AI and machine learning algorithms are supposed to help improve the accuracy of imaging diagnoses and evaluate the regional heterogeneity within a single GBM tumor lesion.

Anti-angiogenic therapy with bevacizumab is a widely used therapeutic agent for recurrent GBM. Radiomics have been demonstrated to be able to improve predicting accuracy for survival and treatment response in recurrent GBM patients treated by bevacizumab [91, 93, 94, 96]. However, the increase of enhancement intensity due to bevacizumab treatment did not necessarily mean tumor response. The change of enhancement intensity could reflect a fluctuation in the permeability properties of the blood brain barrier and the switch of the tumor growth pattern from an infiltrative non-enhancing phenotype to an enhancing one. Even though advanced imaging tools to evaluate cellularity, blood flow hemodynamics, and biochemistry have been developed to solve this issue [111], it remains a challenge to introduce methods to predict tumor response more accurately.

The aggressiveness of GBM is partly due to tumor hypoxia and angiogenesis, which could be assessed by multimodal imaging [112]. Invasive tumors have been revealed to present a different genomic and metabolic abnormalities, which result in a more aggressive GBM phenotype [113]. Attempts have been made to explore the association between invasive GBM imaging-phenotype and genomic abnormalities. For instance, the *MYC* gene was revealed to be related to imaging-phenotypes of deep white matter tracts and ependymal invasion in GBM patients, who had a poor overall survival. Recent radiogenomic studies also indicated that radiomic features related to overall survival of GBM patients were associated with genomic pathways referred to cellular functions, tumor proliferation, immune regulation, and treatment responses [35].

Artificial intelligence (AI) has been applied to traditional and advanced MRI in neuro-oncology to identify infiltrating margins of diffuse gliomas, discriminate pseudoprogression from true progression, and predict tumor recurrence and survival in clinical practice [81]. AI-based radiomics and radiogenomics will benefit noninvasive sampling of tumor microenvironment with high spatial resolution and illuminate underlying heterogeneity of cellular and molecular processes. These tools have also contributed to noninvasive detection of genomic mutations and epigenetic inheritances, for instance *IDH* and *MGMT* genes [34]. Hence, these tools have the potential to more precisely diagnose GBM patients and enable more personalized treatments. Hopefully, applications of AI-based radiomics and radiogenomics is beneficial to diagnosing primary tumor, grading, mutation status and aggression, as well as predicting treatment response and recurrence in neuro-oncology [114].

### Limitations

Gliomas are the most common and aggressive brain tumors, with short survival at the advanced stage [115]. MRI is widely used for the diagnosis of gliomas, but a redundant time needed for manual segmentation of MRI impairs its application as a precise quantitative measurement in the clinical practice. Hence, automatic segmentation is required. However, the large spatial and structural variability among MRI from gliomas patients could be a potential challenge for automatic segmentation.

Machine learning has been applied to medical images as a technique for recognizing patterns [116]. However, metrics for evaluating the performance of machine learning algorithms can result in misleading judgements due to their intrinsic pitfalls. For instance, variance of radiomic textural features derived from MRI involved with can be substantial due to the choice of pulse sequence and other parameters [117], which is worthy of attention especially when combining MRI-derived radiomic features from multicenter patients. Significant variability was observed in radiomic texture features with variations in MRI sources emphasizing the demand for standardized MRI data [118]. In addition, the choice of MRI parameters may not significantly affect the phenotypes of texture analysis, but the spatial resolution may deserve special attention.

Radiomic textural features derived from MRI are found to be sensitive to variations in signal-to-noise ratio and spatial resolution, which forms an obstacle for the clinical application of MRI texture-based prediction in neuro-oncology [119]. Texture features are increasingly sensitive to increasing variations in spatial resolution. However, if the spatial resolution is high enough, the effect of variations in MRI parameters on the accuracy of pattern discrimination almost disappeared. Almost no textural features were robust under spatial resolution variances, except entropy [120]. Hence, relatively unified standards should be established to promote the application of textural features of oncological images as imaging biomarkers in clinical practice beyond the specific tumor type.

The application of AI- and machine learning-based radiomics and radiogenomics is usually limited by access and transparency to research data, such as data ownership, patient privacy and confidentiality. Recent efforts have been made to facilitate applications of radiomics and radiogenomics in medicine and healthcare, especially in medical imaging. For instance, MI2RLNet, an open platform, allows users to share source code and various pre-trained weights for models to boost machine learning-based radiomic research in radiology [121]. MEDAS is another open-source platform, which implements tools in pre-processing, post-processing, augmentation, visualization, and other analyses in radiomics [122]. Studierfenster offers a wide range of capabilities, including the visualization of medical data (CT, MRI, etc.) in two-dimensional (2D) and three-dimensional (3D) space, manual slice-by-slice outlining of structures, and more sophisticated functions involving in convolutional neural network (CNN) [123].

Although AI and machine learning has been applied to facilitate the data mining and model construction in glioma prediction and prognosis, common limitations and pitfalls of machine learning-based radiomics in neuroradiology are worthy of attention, such as selection bias, overfitting and underfitting [124, 125].

AI-based radiomic features have been demonstrated to be qualified for patient stratification in GBM [126, 127]. To reflect spatial and molecular heterogeneity via invasive biomarkers, these AI-based radiomic and radiogenomic features have the potential to classify GBM patients into more precise pre-treatment subgroups and promote better personalized medicine [81]. In addition, all kinds of AI and radiomics derived from conventional and advanced MRI techniques are utilized to differentiate brain tumors from non-neoplastic lesions, discriminate gliomas from lymphomas and metastasis, as well as predict the grading, treatment response and prognosis of gliomas [128]. Radiogenomics further analyze the connection of the radiomic features of the tumor to its microenvironment. Although substantial obstacles still exist, radiologists are ready to introduce AI-based radiomics and radiogenomics into future clinical practice.

### Future directions

Radiomics and radiogenomics are exciting fields with growing applications for CNS neoplasms, but with several challenges warranting further investigation. Challenges related to genomics include the continued requirement for invasive tissue sampling for gold-standard diagnosis, spatial heterogeneity of genomic alterations, variable availability of genetic testing, variable standardization of testing techniques, testing cost issues, and evolving knowledge regarding the relevance of several of the tested genetic alterations. The creation of large public oncological data repositories such as The Cancer Genome Atlas/The Cancer Imaging Archive (TCGA/TCIA) and large-volume data from individual institutions have aided the successful application of AI in glioma. One of the important hurdles in developing neuroimaging or radiogenomic markers for primary brain tumors is the significant variability in image acquisition. Such variability can reflect differences in magnet design by various manufacturers, variable field strength, and differences in the conventional and advanced MRI protocols including pulse sequences, acquisition parameters, acquisition planes, timing of contrast, contrast agent, etc. These features result in differences in the image contrast, spatial resolution, and signal-to-noise ratio (SNR) across different patients or across serial examinations performed in the same patient. With this in mind, a multigroup consensus recommendation for Brain Tumor Imaging Protocol (BTIP) was published to standardize response assessment in glioblastomas (GBMs) in multicenter clinical trials [129, 130]. For developing robust imaging genomics, such standardization is highly important. Despite these challenges, several genetic alterations have already been integrated into the 2021 WHO updated CNS tumor classification; undoubtedly, more will find their way in the future updates. As outlined in this review, there are several useful radiomics features that have shown strong correlation to genomics and appear promising for preoperative prognostic and treatment counseling. This has ushered a new era of research particularly utilizing artificial intelligence for noninvasive prediction of the genetic and biological status of primary brain tumors radiogenomics. Like for genomics, cross-institutional standardization and collaboration is required to achieve the full potential of radiomics.

AI integrated with radiomics and radiogenomics has introduced new perspectives in characterizing noninvasive biomarkers for prediction of survival and tumor recurrence in gliomas, especially HGG patients [44], which therefore promoting treatments tailored to personalized medicine. MRI-based textual analysis also helps characterize regional genetic heterogeneity in GBM, which provides potential diagnostic value under the paradigm of personalized medicine [131]. Machine learning-based radiogenomics directly associate the clinical imaging phenotypes of GBM with underlying morphologic and physiological features [132].

Radiomics and radiogenomics has the potential to significantly improve GBM management via personalized medicine; however, the application of radiomics for GBM treatment remains in developing, since standardized image acquisition and data extraction techniques, as well as larger sample sizes, are needed for constructing machine learning models that are reliable for clinical practice [133]. Furthermore, a lack of basic infrastructures, such as shared software algorithms, architectures, and the tools required for computing, comparing, evaluating, and disseminating predictive models, has hampered the development of radiomics and radiogenomics as well as their clinical implementations. Fortunately, more and more researchers are working to solve these problems. IBEX, an open infrastructure software platform, has been developed to flexibly support common radiomics workflow tasks including multimodality image data import and review, development of feature extraction algorithms, model validation, and consistent data sharing among multiple institutions [134]. Similarly, RayPlus, as a web application, provides multiple functions such as multimodality image import and viewing, ROI definition, feature extraction, and data sharing among multi-institution and multi-department collaborative radiomics research [135]. The quantitative image feature pipeline (QIFP), an open-source, web-based, graphical user interface of quantitative image-processing pipelines for both 2D and 3D medical images, allows users to process and analyze images with no need of self-programming [136]. The QIFP also allows users to access publicly available datasets (e.g., TCIA) through direct links.

## Conclusions

Rather than replace radiologists in clinical practice, AI-based radiomics and radiogenomics aim to provide aid in diagnosis and prediction with higher accuracy and be beneficial to less invasive and more personalized treatment strategies, especially for neuroradiology and to ultimately optimize patient care. In order to continue evolving and make its way into clinical practice, it is critical to establish a general scheme of standardized and reproducible methods for data extraction, analysis, and interpretation, as well as to promote open-access platforms for prospective large-scale multi-center studies. Hopefully, radiomics and radiogenomics will develop to maturation and be widely used for precise medicine in the near future.

### Electronic supplementary material

Below is the link to the electronic supplementary material.

Supplementary Material 1

## Data Availability

The datasets used and analyzed during the current study are available from the corresponding author on reasonable request.

## Abbreviations

CNS: Central Nervous System
GBM: Glioblastoma
HGG: High Grade Glioma
LGG: Low Grade Glioma
MRI: Magnetic Resonance Imaging
T1W: T1-weighted
CE: Contrast-Enhanced
T2W: T2-weighted
FLAIR: Fluid-Attenuated Inversion Recovery
ROIs: Region of Interests
TCIA: The Cancer Imaging Archive
TCGA: The Cancer Genome Atlas
CCGA: The Chinese Glioma Genome Atlas
IDH: Isocitrate Dehydrogenase
VASARI: The Visually Accessible Rembrandt Images
AI: Artificial Intelligence
CNN: Convolutional Neural Network
SVM: Support Vector Machine
