# Supplementary material for: Artificial intelligence-based MRI radiomics and radiogenomics in glioma
Source: Cancer Imaging. 2024 Mar 14;24:36. doi: 10.1186/s40644-024-00682-y (PMC10938723; doi:10.1186/s40644-024-00682-y)
Supplement: Supplementary file 1 — Supplementary Material 1 [file 40644_2024_682_MOESM1_ESM.docx]

**Rebuttal Letter**

Dear editors and reviewers,

We have carefully revised the manuscript entitled “**Artificial intelligence-based MRI radiomics and radiogenomics in glioma**” according to the comments of the editors and reviewers*.* There is no conflict of interest in the submission of this revised manuscript, and all authors have reached consensus for publication in ***Cancer Imaging***. On behalf of my co-authors, I would like to declare that this manuscript has not previously been published in whole or in part. All listed authors have approved the attached manuscript.

We hope that this work will be accepted by ***Cancer Imaging***. We are very grateful for your consideration of our manuscript and look forward to further responses from the reviewers.

Yours sincerely,

Dr. Xin Liao

**COMMENTS FROM REVIEWERS**

**Reviewer #1:** All my questions are well addressed. However, the reference numbers seems to be not corrected yet (e.g. PMID: 34185076). Please update the number of references. Otherwise the overall quality is much improved.

Thank you for your correction.

**Reply to Q1:** Dear reviewer, the reference numbers have been corrected and updated in the revised manuscript. Thanks for your comments.

**Reviewer #2:** This is an interesting topic and the revisions satisfactorily address the prior reviewer comments. I believe the article is acceptable.

**Reply to Q1:** Dear reviewer, thanks for your comments.
